# Scrub Typhus Presenting as Acute Flaccid Paralysis in a Child: A Differential to Be Included in a Common Presentation

**DOI:** 10.7759/cureus.27909

**Published:** 2022-08-11

**Authors:** Pradeep Kumar, Arun Prasad, Subhash Kumar, Ambrin Akhtar

**Affiliations:** 1 Department of Pediatrics, All India Institute of Medical Sciences, Patna, IND; 2 Department of Radiodiagnosis, All India Institute of Medical Sciences, Patna, IND

**Keywords:** doxycycline, afp, meningoencephalitis, muscle weakness, ivig

## Abstract

Neurological manifestations are common in scrub typhus in children. A 12-year-old girl presented with acute onset fever, bilateral lower limb weakness, and urinary retention. On initial investigations, scrub typhus immunoglobulin M (IgM) ELISA (enzyme-linked immunosorbent assay) was reactive. She was given an injection of doxycycline along with other supportive therapies. Her symptoms improved gradually and bilateral lower limb power came back gradually without residual weakness by the 13th day of admission.

## Introduction

Scrub typhus is an acute zoonotic disease caused by *Orientia tsutsugamushi* and transmitted by trombiculid mites [1]. Historically scrub typhus was described in Japan in the 1800 AD, and then in Japan and Malaysia in the early 20th century [2]. It is a serious public health problem in the Asia-Pacific region. As per the current estimates, it threatens one billion people globally and leads to illness in around one million people every year [3]. A high index of suspicion is required to diagnose scrub typhus infections, as the clinical features are often confused with dengue, malaria, leptospirosis, meningoencephalitis, and viral infections. Untreated cases often have a high case fatality. Bonell et al., in a systematic review, reported varying mortality reports with a median mortality of 6.0% and 1.4% for untreated and treated scrub typhus respectively. Existing evidence suggests high mortality in complicated scrub typhus with CNS (central nervous system) involvement (13.6% mortality) and multi-organ dysfunction (24.1%) [4]. Delayed treatment with doxycycline has been associated with major organ dysfunction as well as prolonged hospitalization, emphasizing that it is imperative to start early empirical doxycycline therapy in suspected cases [5].

Meningoencephalitis is the most commonly reported neurological manifestation of scrub typhus [6]. Overall, the neurological complications of scrub typhus include aseptic meningitis, meningoencephalitis, seizures, delirium, hearing loss, cerebellitis, and myelitis [7]. In scrub typhus, bacteria enter from the periphery to the central nervous system via a hematogenous route. *Orientia tsutsugamushi* is frequently seen in circulating mononuclear cells. There is prolonged microbial survival in leukocytes and phagocyte-facilitated infection may cause an invasion of the central nervous system [8].

Few cases of scrub typhus with GBS (Guillain-Barré syndrome) have been reported in adults [9]. We report a case of scrub typhus with meningoencephalitis who developed acute flaccid paralysis in the form of paraparesis, which improved gradually with an injection of doxycycline and other supportive therapies. Paraparesis resolved completely in two weeks and no residual weakness was noted on subsequent follow-up.

## Case presentation

A 12-year-old female child was admitted with complaints of fever of 102°- 104° F for four days and muscle weakness in both lower limbs for three days with a history of urinary retention without any sensory involvement. The weakness of all groups of muscles in bilateral lower limbs developed simultaneously. The patient did not specifically appreciate any difference in the involvement of lower limb muscle groups. There was no history of ear discharge, trauma to the head or spine, contact with tuberculosis, recent intramuscular injection, snake bite, poisoning, or history of travel, outdoor camping, or playing in the forest. Rash was absent and we found no eschar after extensive search especially in the armpit, groin as well as exposed areas. Her developmental milestones were as per age. At the time of admission, the patient was conscious, oriented, and febrile, with a temperature of 102°F. Her CNS findings were a Glasgow coma scale of 15/15, hypotonia in bilateral lower limbs, 0/5 power in bilateral lower limbs, absent knee and ankle reflexes, and abdominal and plantar reflexes. Her Hughes grade was 4 and her MRC (Medical Research Council) sum score was 30/60 [10,11]. Sensory and autonomic examination revealed no problem. The total MRC sum score ranges from zero (total paralysis) to 60 (normal strength). The score is the sum of the MRC score of six muscles (three at the upper and three at the lower limbs) on both sides, each muscle graded from zero to five. The following muscles were examined: Deltoid, Biceps, Wrist extensor, Iliopsoas, Quadriceps femoris, and Tibialis anterior (Tables 1, 2).

**Table 1:** Hughes’ grade with subdivision of grades Reference no. [10]

Grades	Functions
Grade 0	Normal functional state
Grade 1	Able to run with minor signs and symptoms
Grade 2	Able to walk 5 meters independently
Grade 3	Able to walk 5 meters with aid
Grade 4	Bed or chair bound
Grade 5	With respiratory failure
Grade 5 A	With early respiratory failure, no requirement for a ventilator
Grade 5 B	Respiratory failure requiring mechanical ventilation
Grade 6	Death

**Table 2:** Medical Research Council (MRC) sum score Reference no. [11]

Grade	Degree of Strength
5	Normal Strength
4	Ability to resist moderate pressure throughout a range of motion
3	Ability to move through a full range of motion against gravity. If a subject has a contracture that limits joint movement, the mechanical range will be to the point at which the contracture causes joint restriction
2	Ability to move through a full range of motion with gravity eliminated
1	A flicker of motion is seen or felt in the muscle
0	No movement

She was admitted to PICU (pediatric intensive care unit) and appropriate investigations were sent to establish the cause of fever and acute flaccid paralysis (AFP). Local AFP surveillance team informed as per unit protocol. IVIg (Intravenous Immunoglobulin) 2 gm/kg was given over five days suspecting AFP due to GBS. Her complete blood count was normal except for hemoglobin, which was on the lower side of normal and mild lymphocytosis. The peripheral smear showed microcytic hypochromic red cells with increased lymphocytes and no hemoparasites were noted. Liver function showed an increase in transaminases. Serum electrolytes were normal with serum calcium slightly in the lower range. Her C-reactive protein, creatinine phosphokinase, and serum ammonia were increased (Table 3).

**Table 3:** Laboratory investigations PCV: packed cell volume, MCV: mean corpuscular volume, ESR: erythrocyte sedimentation rate, CPK: creatinine phosphokinase

Investigation	Normal value	Day 1	Day 6	Day 8
Hemoglobin (g/dL)	11.5–14.5	11	9.7	
Platelets (per cmm)	150–450×10^3^	150×10^3^	179×10^3^	
Leucocyte count (per cmm)	4,000–11,000	11,400	8,310	
Differential leucocyte count (%):				
Neutrophils	40-80	39.9	59.1	
Lymphocytes	20-40	57.6	35.4	
Monocytes	2-10	1.8	2.0	
Eosinophils	1-6	0	2.1	
Basophils	0-1	0.7	0	
PCV (%)	35-45	83.7	87.6	
MCV (fL)	78-95	13.5	13.9	
ESR (mm in first hour)	0-10	70	43	
Total serum bilirubin (mg/dL)	0.3–1.2	0.83		0.59
Serum direct bilirubin (mg/dL)	<0.2	0.26		0.25
Aspartate aminotransferase (IU/L)	<31	125.3		83.8
Alanine aminotransferase (IU/L)	10–28	69.5		137.1
Alkaline phosphatase (IU/L)	100–290	103.5		112.9
Total protein	6.0-8.0	6.90		
Albumin/Globulin (gm/dL) ratio		0.84		0.63
Blood urea (mg/dL)	13–43	27.5		20.6
Serum creatinine (mg/dL)	0.7–1.3	0.47		0.37
Serum sodium (mmol/L)	135–145	129.2		132.7
Serum potassium (mmol/L)	3.5–5	4.4		4.2
Serum calcium (mg/dL)	8.8-10.8	8.2		8.4
Serum phosphate (mg/dL)	3.2-5.8	3.5		5.2
C-reactive protein (mg/L)	0-5	29.2	12.8	
CPK (IU/L)	20-180	256.78		200.32
Serum ammonia (µmol/L)	11-32	394		

HIV I & II (Human Immunodeficiency Virus) antibodies, HBsAg (Hepatitis B surface antigen), and Anti-HCV (Hepatitis C Virus) antibodies were non-reactive. Leptospira serology was non-reactive. Dengue NS1 (Non-structural Protein 1) antigen, IgM (Immunoglobulin M), and IgG (Immunoglobulin G) were negative but scrub typhus IgM ELISA (enzyme-linked immune sorbent assay) was reactive (Table 4).

**Table 4:** Serology reports NS1: non-structural protein 1, IgM: immunoglobulin M, IgG: immunoglobulin G, ELISA: enzyme-linked immune sorbent assay, HIV: human immunodeficiency virus, HBsAg: hepatitis B surface antigen, HCV: hepatitis C virus

Tests	Reports
Malaria card test	Negative
Dengue NS1 Antigen, IgM, IgG	All non-reactive
Leptospira IgM	Non-reactive
Scrub typhus IgM ELISA	Reactive
HIV I & II antibody	Non-reactive
HBsAg	Negative
Anti HCV antibody	Non-reactive
Widal test	Negative

CSF (cerebrospinal fluid) on day one showed 80% lymphocytes with 20% polymorphs and increased LDH (lactate dehydrogenase), the rest of the parameters were normal (Table 5).

**Table 5:** CSF examination reports on different stages of illness CSF: cerebrospinal fluid, AFB: acid-fast bacilli, CBNAAT: Cartridge based nucleic acid amplification test, ADA: adenosine deaminase, LDH: lactate dehydrogenase

Parameters	Normal value	Day 1	Day 9
Volume		3ml	3ml
Colour	Colorless	Colorless	Colorless
Appearance	Clear	Clear	Clear
Cells (mm3)	<5	20	2
Lymphocytes	≥75%	80%	100%
Polymorphs	0	20%	
Gram staining	Negative	Negative	Negative
AFB staining	Negative	Negative	Negative
CBNAAT	Negative	Negative	Negative
Culture and sensitivity	No growth	No growth	No growth
Protein (mg/dL)	20-45	57.55	36.10
Sugar (mg/dL)	>50 (or 75% serum glucose)	59	70.04
ADA (IU/L)	< 10	2.76	< 0.10
LDH (IU/L)	< 40	89.85	33.95

**Figure 1:**
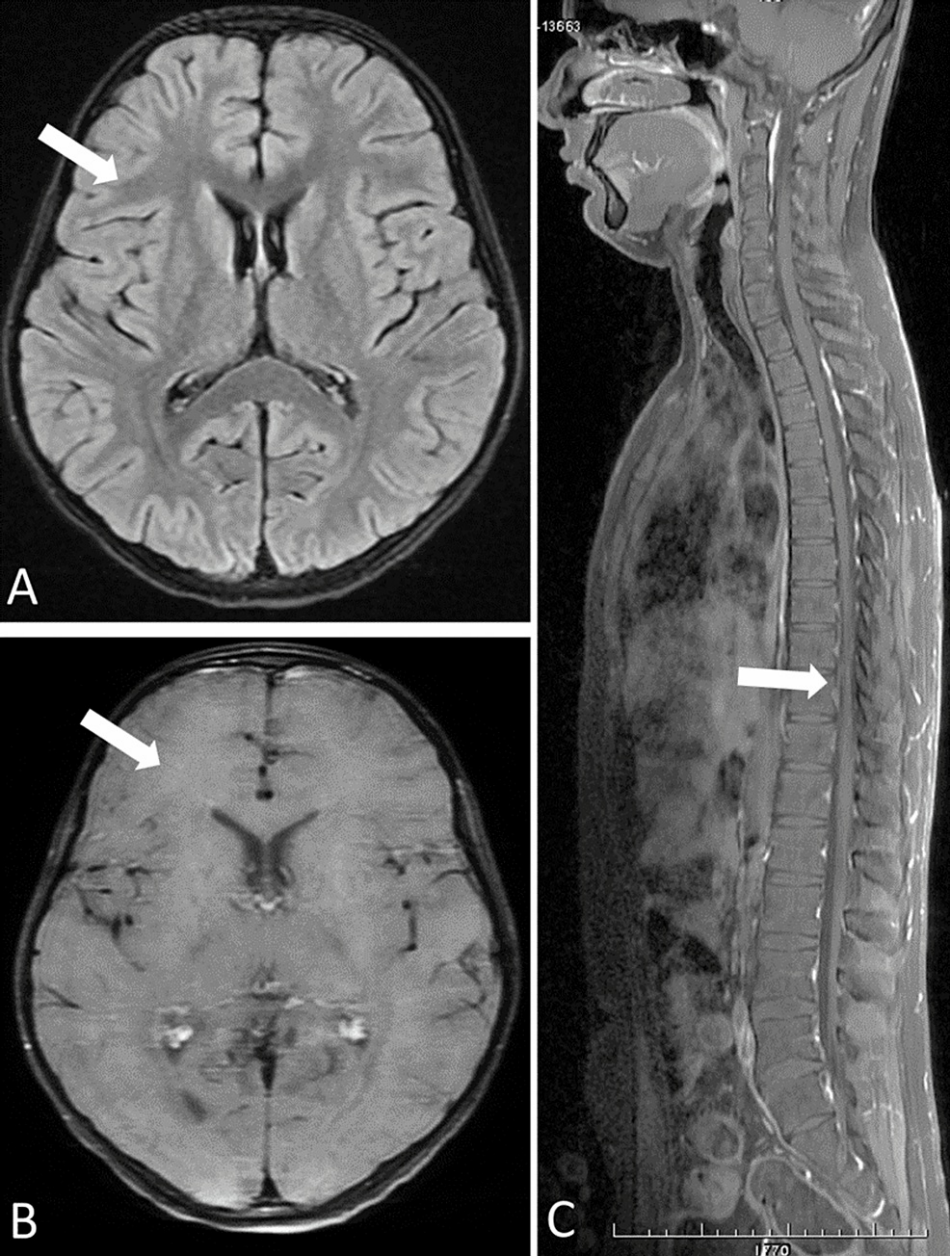
MRI brain and spine MRI (Magnetic Resonance Imaging) brain and spine A) Axial FLAIR (fluid-attenuated inversion recovery) image of brain showing normal appearance of the brain parenchyma (white arrow) B) Axial post-contrast T1-FS image of the brain showing the normal appearance, without any abnormal meningeal or parenchymal enhancement (white arrow) C) Sagittal post-contrast T1-FS image of the whole spine showing the normal appearance, without any abnormal meningeal or spinal cord enhancement (white arrow)

Intravenous (IV) doxycycline was started for scrub typhus. Other supportive therapies like IV paracetamol for fever and intravenous fluids were also started. We had initially started 3% saline considering raised intracranial pressure as meningoencephalitis is the most common neurological involvement, which was later stopped in view of no clinical or CSF features suggestive of the same. Nerve conduction velocity was also done which was normal. The patient developed warm shock on the next day, hence noradrenaline was started. Blood culture showed no growth. CSF study was repeated on day nine of illness and the reports were normal on all parameters. Her MRI (Magnetic Resonance Imaging) brain and spine were reported to be normal (Figure 1).

X-ray chest was also normal. Routine examination of urine did not show any abnormality. The patient gradually improved with an injection of doxycycline and other supportive therapy. She recovered from warm shock gradually and her power in bilateral lower limbs improved from 0/5 to 2/5 on day four, 3/5 on day nine, and 5/5 by day twelve, and discharged on day 13 with no residual weakness (Table 6).

**Table 6:** Improvement in neurological parameters MRC: medical research council, UL: upper limbs, LL: lower limbs

Neurological parameters	Day1	Day 4	Day 9	Day 13
MRC sum score	30/60	38/60	54/60	60/60
Hughes’ score	4	4	3	0
Muscle power UL	5/5	5/5	5/5	5/5
Muscle power LL	0/5	2/5	3/5	5/5
Reflexes	Areflexia	Areflexia	Normal	Normal

## Discussion

Scrub typhus is an important cause of acute febrile illness and is rampant in Asian countries. It is reported to have wider distribution across South America and Africa [4]. It has a whole gamut of neurological manifestations. Meningoencephalitis is one of the commonest neurological manifestations [6]. We found reports of acute transverse myelitis and GBS more common in adults than in children. We found one case report of exclusive paraparesis in children after an extensive search of published literature to date.

Gangula et al. reported GBS in a 40-year-old male patient with scrub typhus and *Plasmodium falciparum* infection simultaneously [9]. Dev et al. have reported GBS in a 20 years old male patient who had concurrent scrub typhus and leptospira infection as well [12]. In our case, we could not establish GBS, as there was no involvement of the upper limbs suggestive of ascending paralysis. Also, there was no albumin-cytological dissociation in the second CSF examination during the second week of illness and the nerve conduction velocity was normal too.

Ryu et al. found paraparesis in a 66 years old patient who was found to have eschar, the motor weakness recovered completely in five days of steroid pulse therapy and seven days of injectable doxycycline, but urinary retention persisted even after one year of follow-up [13]. Lee et al. reported acute transverse myelitis in a 54-years-old male patient suffering from scrub typhus with eschar and was controlled voiding on alpha-adrenergic blockers [14]. Tandon et al. in 2022 reported long‑segment myelitis, meningoencephalitis, and axonal polyneuropathy in a 17-year-old adolescent who had co-infection of neurocysticercosis. The authors report residual weakness after three months of follow-up [15]. 

Muranjan and Karande reported a case of acute paraparesis due to lumbosacral radiculopathy with concomitant meningitis in a 13-month-old child who recovered completely without residual paraparesis in the second month of illness [16]. In our patient, who was a 12-year-old girl, we found evidence of meningitis with normal neuroimaging of the brain and spine. The reported literature suggests residual weakness in most cases of paraparesis in adults and adolescents, our patient had clinical AFP involving bilateral lower limbs, which recovered fully by the end of the second week of illness.

## Conclusions

Scrub typhus should also be considered in the differential diagnosis of acute flaccid paraparesis, especially when it is associated with an altered mental state, or clinical and CSF-proven meningoencephalitis in rickettsia endemic areas. It is a fatal disease but responds well to the available medications (doxycycline or azithromycin). Most of the cases recover well without residual weakness. Fatality reduces significantly if prompt treatment is started before the development of hypotensive shock.
